# Strategies to control therapeutic antibody glycosylation during bioprocessing: Synthesis and separation

**DOI:** 10.1002/bit.28066

**Published:** 2022-02-28

**Authors:** Elizabeth Edwards, Maria Livanos, Anja Krueger, Anne Dell, Stuart M. Haslam, C. Mark Smales, Daniel G. Bracewell

**Affiliations:** ^1^ Department of Biochemical Engineering University College London London UK; ^2^ Department of Life Sciences Imperial College London London UK; ^3^ School of Biosciences, Division of Natural Sciences University of Kent Canterbury UK; ^4^ National Institute for Bioprocessing Research and Training Foster Avenue, Blackrock Dublin Ireland

**Keywords:** antibodies, biosimilars, CQAs, downstream glycosylation bioprocessing, mAbs, *N*‐glycosylation

## Abstract

Glycosylation can be a critical quality attribute in biologic manufacturing. In particular, it has implications on the half‐life, immunogenicity, and pharmacokinetics of therapeutic monoclonal antibodies (mAbs), and must be closely monitored throughout drug development and manufacturing. To address this, advances have been made primarily in upstream processing, including mammalian cell line engineering, to yield more predictably glycosylated mAbs and the addition of media supplements during fermentation to manipulate the metabolic pathways involved in glycosylation. A more robust approach would be a conjoined upstream–downstream processing strategy. This could include implementing novel downstream technologies, such as the use of Fc γ‐based affinity ligands for the separation of mAb glycovariants. This review highlights the importance of controlling therapeutic antibody glycosylation patterns, the challenges faced in terms of glycosylation during mAb biosimilar development, current efforts both upstream and downstream to control glycosylation and their limitations, and the need for research in the downstream space to establish holistic and consistent manufacturing processes for the production of antibody therapies.

## Graphical abstract

Upstream and downstream processing strategies to control antibody glycosylation are reviewed. Edwards et al. have presented various approaches in both upstream and downstream processing to direct antibody glycosylation towards certain profiles. Upstream examples include cell line engineering, media supplementation, and manipulating bioreactor conditions. Downstream techniques have also been developed such as the use of Fc γ‐receptor 3a affinity ligands for glycovariant separations, but the authors have highlighted that there is notably less development in this area.

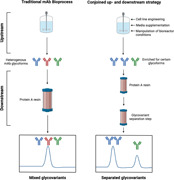

## 1 INTRODUCTION

Out of all known posttranslational modifications, glycosylation has one of the most significant impacts on therapeutic antibody pharmacokinetics (Boune et al., 2020). Glycosylation of antibodies changes as a result of aging, immune events such as infections and environmental factors. Such changes have been associated with autoimmune and inflammatory diseases (Hashii et al., 2009; Kemna et al., 2017). Glycosylation is known to have effects on the biological activity, solubility, serum half‐life, and safety of therapeutic antibodies (Bas et al., 2019; Narhi et al., 1991; Varki, 1993). Glycans have an important role in immunity and self‐recognition during common immune events and, ultimately, can impact the therapeutic efficacy of biopharmaceuticals (Gagneux & Varki, 1999; van Kooyk & Rabinovich, 2008). Therefore, controlling glycosylation in antibody biotherapeutics is of critical importance.

**Figure 1:**
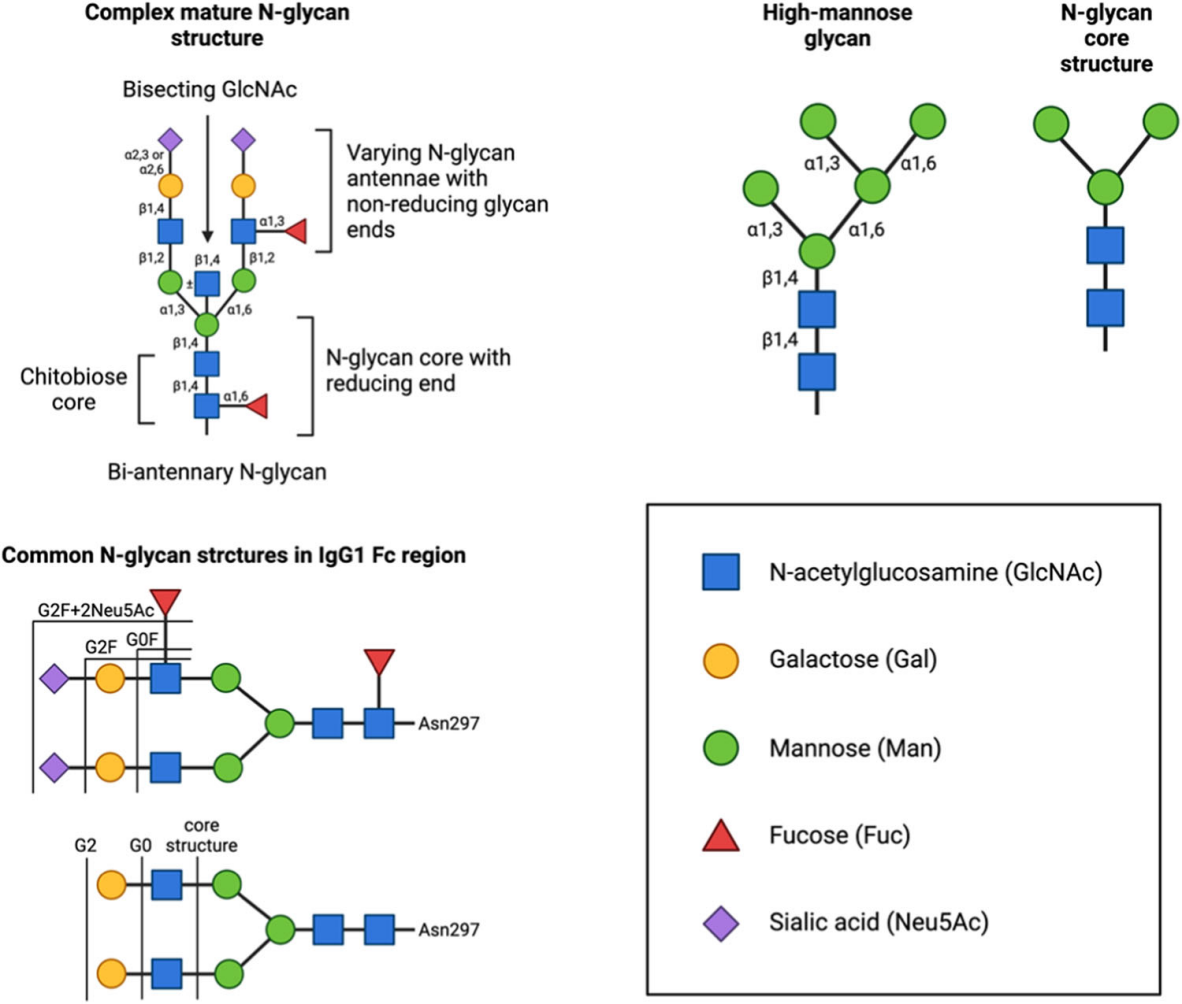
Simplified *N*‐glycan structure scheme showing the complexity of matured biantennary *N*‐glycan structures, general *N*‐glycan nomenclature and common *N*‐glycan structures seen attached to Asn297 in the Fc region of IgG1. Reducing and nonreducing terminology is applied from basic glycobiology. Bisecting *N*‐glycans are common in human serum. Complex *N*‐glycans follow a complex core structure comprising five sugar residues, three mannoses, and two *N*‐acetylglucosamine (GlcNAc) residues. The immature *N*‐glycan is normally trimmed back until the core structure is created to achieve full complexity of the maturing *N*‐glycan required for monoclonal antibodies (mAbs). *N*‐glycans in general can reach higher branching

A good example of the impact of glycosylation on a biotherapeutic protein is that of intravenous immunoglobulin (IVIg), which is the treatment of choice for patients with immunodeficiencies and inflammatory diseases such as Kawasaki disease, dermatomyositis and lupus. It is prepared from pools of plasma‐derived IgG, which are harvested from tens of thousands of donors to capture a diverse antibody repertoire (Jolles et al., 2005). Despite IVIg being a standard treatment for several different diseases, and these preparations having been used for over 30 years, their precise mechanism of action is yet to be elucidated. However, it has been concluded that the presence of terminal sialic acid residues (see Figure 1) on the Fc glycan of IgG is crucial to the clinical efficacy of IVIg in the context of many different disease models (Anthony & Ravetch, 2010; Anthony et al., 2008; Brückner et al., 2017; Schwab et al., 2014). Among other observations, desialylation of the Fc fragment reduces the anti‐inflammatory activity of IVIg. Conversely, the enrichment of these preparations with sialic acid results in improved in vivo efficacy in a mouse model of rheumatoid arthritis (Käsermann et al., 2012). Interestingly, IgG glycans and other serum glycoproteins have been shown to vary with age (de Haan et al., 2016; Merleev et al., 2020; Štambuk et al., 2020). Age‐dependent Fc‐glycosylation may be relevant to the generation of biotherapeutics for more severe disease types. Similarly, customized medicines in children, pregnant women or other immune sensitive groups may benefit from a more finely tuned and less heterogenous glycosylation profile.

**Figure 2:**
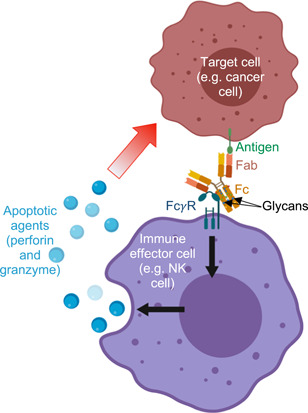
Monoclonal antibody (mAb) mechanism of action by way of antibody‐dependent cellular cytotoxicity (ADCC). The antigen binding (Fab) region of the mAb binds the antigen on the surface of a target cell, such as a cancer cell. The Fc region then binds an Fc γ‐receptor (Fc γR) on the surface of an immune effector cell, such as a natural killer (NK) cell. This cross‐linking stimulates the NK cell to release apoptotic agents that kill off the cancer cell in a targeted manner. IgG are glycosylated in the Fc region and sometimes also in the Fab region, and these glycans mediate clinically relevant properties such as serum half‐life, pharmacokinetics, target binding affinity, and overall therapeutic efficacy. Created with BioRender.com

**Figure 3:**
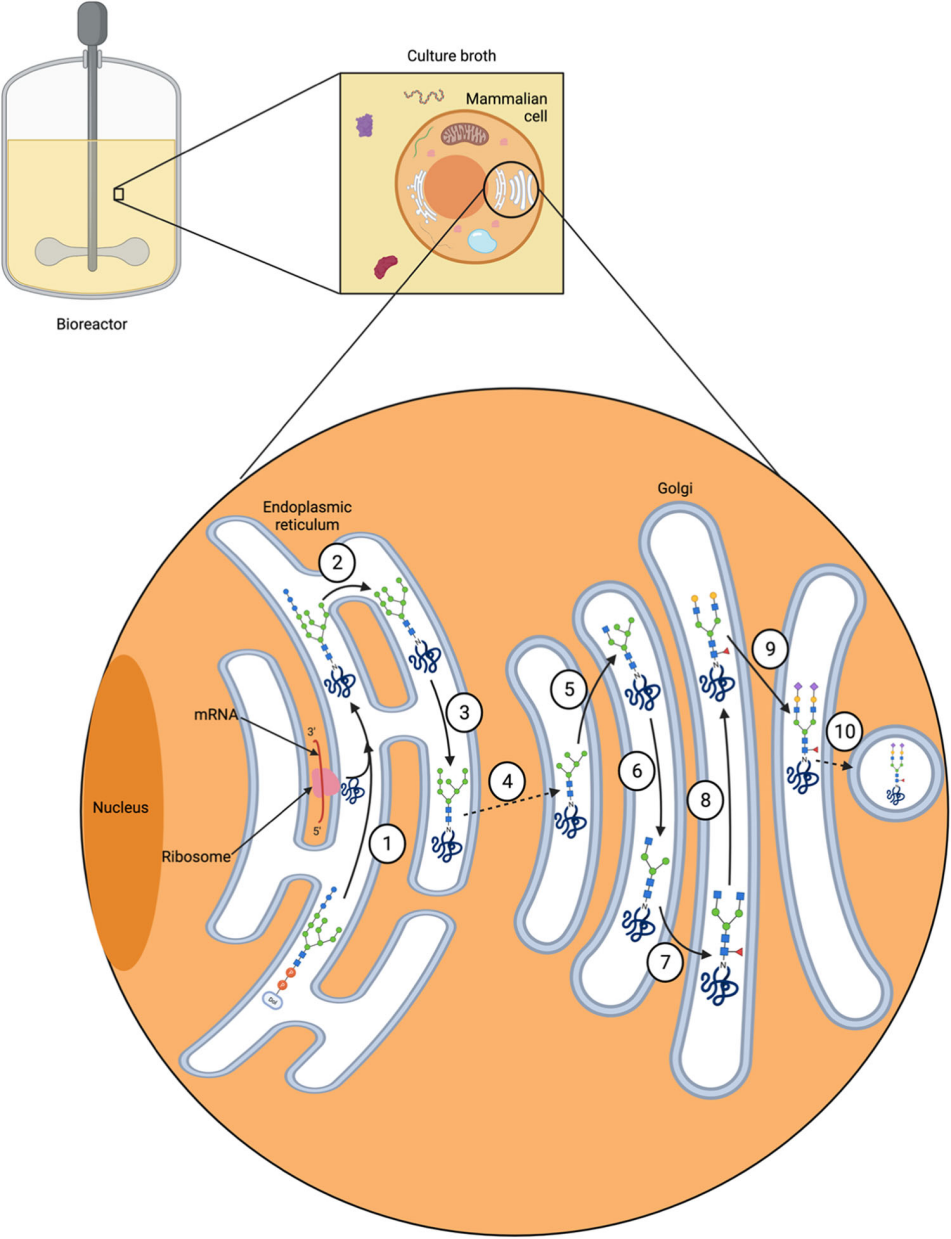
Schematic of the monoclonal antibody (mAb) *N*‐glycosylation pathway that occurs during mammalian cell culture and the possible stages at which heterogeneity can arise. (1) an immature precursor *N*‐glycan (three glucose, nine mannose, and two *N*‐acetylglucosamine residues, Glc_3_Man_9_GlcNAc_2_) is transferred from the dolichol phosphate anchor onto an asparagine (Asn) residue on the nascent mAb protein within an Asn‐X‐Ser/Thr, except Pro motif within the primary protein structure. (2) The three terminal Glc residues are further cleaved by a set of glucose specific glucosidases. (3) The immature *N*‐glycan is further trimmed to an immature Man8 *N*‐glycan structure. (4) The immature glycoprotein is transported from the endoplasmic reticulum (ER) to the Golgi apparatus (Golgi) via vesicular trafficking. Further trimming to a Man5 structure occurs in the Golgi. (5) ββ‐1,2‐*N*‐acetylglucosaminyltransferase I adds a GlcNAc residue to one of the terminal mannose residues. (6) Two terminal mannose residues are trimmed by α‐mannosidase II. (7) Another *N*‐acetylglucosyltransferase adds a GlcNAc residue onto the terminal mannose residue, followed by a fucosyltransferase adding a fucose sugar to the core GlcNAc. (8) Two galactose (Gal) residues are added to the two terminal GlcNAcs by specific galactosyltransferases. (9) Two sialic acid residues are added to the two terminal Gals by specific sialyltransferases and, finally, (10) the glycoprotein is encased in a vesicle, which buds off from the Golgi and travels towards the cell surface. There are about 100 glycosyltransferases and glucosidases involved in the processing and trimming of *N*‐glycans and other glycoconjugates. Most biosynthetic steps are precursor‐dependent, which gives rise to an immense variety of glycans described as heterogeneity. Created with BioRender.com

Despite the ubiquity and importance of glycosylation, control of *N*‐ and *O*‐linked glycosylation remains a challenge in biopharmaceutical manufacturing due to the potential glycosylation heterogeneity of a glycoprotein. This heterogeneity can have an impact on antibody effector functions such as antibody‐dependent cellular cytotoxicity (ADCC; see Figure 2). It arises due to the intricate and complex cellular process by which proteins are glycosylated (see Figure 3). The heterogeneity can be divided into two types: micro‐ and macroheterogeneity. Microheterogeneity refers to the variability of the glycans at each glycosylation site. In recombinant protein production, this primarily occurs as a result of the choice of host cell expression system and the balance of glycosylation enzymes expressed. Other important factors include the metabolism of the host cell, the media composition and any associated feeding regime, pH, and the temperature of the fermentation process. On the other hand, heterogeneity due to the presence or absence of glycans at particular glycosylation sites in the protein primary structure is termed macroheterogeneity (Hossler, 2012).

Overall, IgG antibodies are mainly biantennary *N*‐glycosylated in their Fc‐region at a single site (Asn297). The overall diversity of *N*‐glycosylation lies primarily in the antennae of the glycan and in the presence or absence of a fucose residue on the core of the *N*‐glycan (see Figure 1). However, other glycosylation sites have been observed in serum IgGs in the variable Fab region. Such *N*‐glycans have been shown to have a greater level of structural heterogeneity (Anumula, 2012; Bondt et al., 2014). Further, somatic mutation induces additional Fab *N*‐glycosylation in monoclonal antibodies (mAbs) derived from human synovial tissue B cells from patients with rheumatoid arthritis, with antigen specificity against citrullinated histone. These mAbs showed a variable Fab *N*‐glycan‐dependent antigen binding (Corsiero et al., 2020). Interestingly, B‐cell‐derived, experimentally point‐mutated mAbs (anti‐adalimumab and anti‐infliximab), which had Fab *N*‐glycans deleted in complementarity‐determining regions appeared structurally more thermally unstable compared with their glycosylated wild types (WTs; van de Bovenkamp et al., 2018). However, increased antigen binding induced by Fab‐ *N*‐glycans appears to be antigen‐dependent, as others reported a decrease in CD33‐binding affinity in the presence of Fab *N*‐glycosylation (Co et al., 1993). In summary, the antigen‐binding affinity might be differentially modulated via Fab‐*N*‐glycosylation. However, more investigation of Fab *N*‐glycosylation and modulation of antigen‐binding affinity is required to further understand the binding effects of Fab *N*‐glycosylation regarding different types of antigens.

As glycosylation is diverse and challenging to control during bioprocessing, most US Food and Drug Administration (FDA)‐ or European Medicines Agency (EMA)‐approved IgG‐type biotherapeutics contain *N*‐glycosylation in the Fc region only. Cetuximab, however, is glycosylated in the Fab region, so the glycosylation of both the Fab and Fc regions of this molecule must be monitored (Janin‐Bussat et al., 2013). As glycosylation heterogeneity substantially impacts mAb immunogenicity and therapeutic efficacy, the control and characterization of mAb glycosylation profiles is specified in ICH guideline Q6B (EMA, 1999). The EMA also state that “particular attention should be paid to the degree of sialylation, galactosylation, mannosylation, and fucosylation” (Carillo et al., 2020).

The diversity of IgG glycosylation was demonstrated by Pucić et al. (2011), whereby *N*‐glycan content distribution on three human serum IgG populations was reported for 24 *N*‐glycan structures out of 36 theoretical structures. Of these, 96% of all neutral *N*‐glycan structures contained core fucose. Agalactosylated and monogalactosylated glycan content was similar at around 40% with digalactosylated structures making up the remaining 20%. Only 11.6% of monogalactosylated structures and 50% of digalactosylated were sialylated (Pucić et al., 2011). However, as *N*‐glycans vary with age and location, the optimal *N*‐glycosylation for a given IgG biotherapeutic may depend on the target group and the required biological activity.

Due to the tendency for regulations to become more rigorous over time and as advances are made in this area, we anticipate that the requirement for the control and characterization of mAb glycosylation patterns will become more stringent. Monitoring and control of glycosylation during bioprocessing is therefore paramount. However, techniques for in‐ and online characterization of glycoprofiles are rarely, if ever, reported in industrial‐scale bioprocessing. Further, as is discussed in Section 2.3, during mAb biosimilar development the glycosylation patterns of both the proposed biosimilar and the innovator drug must be characterized and matched as closely as possible. Table 1 details the upcoming patent expiration dates of a number of blockbuster mAbs and antibody‐drug conjugates (ADCs), both in Europe and in the US, illustrating the rich pipeline and potential for biosimilar development over the coming decade (Duivelshof et al., 2019). Attempts to control mAb glycosylation so far have mainly focused on cell line engineering, media supplementation and process parameter alterations, such as temperature and pH shifts during fermentation. Enzymatic approaches to glycoengineering have also been investigated but are not always scalable. In particular, the control of IgG core fucosylation using hydrolytic fucosidases has proved difficult due to the lack of an enzyme that is able to specifically and efficiently remove the core fucose of the Fc N‐glycan on IgG (Tsai et al., 2017). Whilst upstream approaches allow the fine‐tuning of mAb glycosylation and its heterogeneity to an extent, it remains difficult to predict and tightly control such an intricate process. Therefore, to develop bioprocesses that give more control over the ultimate mAb glycosylation patterns, attention should turn to downstream events so that integrated processes which produce mAbs with more defined glycosylation profiles can be developed.

**Table 1:** Blockbuster mAbs and ADCs with upcoming patent expirations in Europe and the United States

mAb	Patent expiration date	
	Europe	United States
Adcetris® (brentuximab vedotin)	August 2023	2015–2031
Avastin® (bevacizumab)	January 2022	July 2019
Campath® (alemtuzumab)	May 2021	July 2021
Cyramza® (ramucirumab)	May 2023	November 2025
Darzalex® (daratumumab)	May 2026	February 2025
Gazyvaro®, Gazyva® (obinutuzumab)	November 2024	January 2035
Kadcyla® (trastuzumab emtansine)	June 2020	September 2026
Keytruda® (pembrolizumab)	June 2028	November 2036
Opdivo® (nivolumab)	May 2026	June 2027
Perjeta® (pertuzumab)	May 2023	June 2024
Prolia®, Xgeva® (denosumab)	June 2022	February 2025
Tecentriq® (atezolizumab)	September 2027	May 2028
Yervoy® (ipilimumab)	2021	2023

*Note*: Obinutuzumab is glycoengineered to have reduced core fucosylation, illustrating the commercial potential of further investment and development in the area of glycoengineered mAbs. Adapted from Busse and Lüftner (2019).

Abbreviations: ADC, antibody‐drug conjugate; mAbs, monoclonal antibodies.

Here we review how different glycosylation patterns of certain therapeutic antibodies affect their safety and clinical efficacies. Advances that have been made in genetic engineering, upstream processing and enzymatic glycoengineering to direct glycosylation towards particular profiles, as well as downstream development that has also been made in this area, are then explored. Finally, future perspectives and developments that could provide a step‐change in the area are described, particularly with a focus on specific areas of downstream processing that could be targeted for developing approaches to control mAb glycosylation patterns. Development of such approaches would allow the separation and purification of IgG therapeutics with specific glycosylation patterns, or more homogenous mAb glycoprofiles, and facilitate experiments to determine if such products are more clinically efficacious.

## 2 *N*‐GLYCOSYLATION PATTERNS OF THERAPEUTIC ANTIBODIES

As different glycosylation patterns affect the clinical efficacies of biopharmaceuticals, platforms must be developed to yield the desired glycosylation patterns on a case‐by‐case basis. Glycosylation in the Fc region affects IgG affinity for Fc γ‐receptors (Fc γRs), which mediate effector functions such as ADCC, phagocytosis and cytokine secretion (Junker et al., 2020). Complement‐dependent cytotoxicity (CDC) is also an important effector function for IgG therapeutics which can be impacted by different glycosylation patterns in the Fc region, particularly galactosylation (Abès & Teillaud, 2010; Reusch & Tejada, 2015; Ząbczyńska et al., 2020). Here we focus on the effect of glycosylation on ADCC, as it is a more common effector function of IgG‐type therapeutics, particularly those used for oncological indications. As of 2020, out of all 22 FDA‐approved antitumor therapeutic antibodies, ADCC was listed as contributing to the mechanism of action for 11 of these drugs, whereas CDC was listed for only 5 (Yu et al., 2020). Interestingly, some patients receiving antibody therapy experience resistance or limited clinical responses. For example, there is both a high‐ and a low‐affinity allotype of the Fc γ‐receptor 3a (Fc γRIIIa), one of the receptors on the surface of natural killer cells that mediate the therapeutic activity of many IgG1‐type mAbs. A patient will have a different response to a mAb therapy depending on which of these allotypes they express. This has resulted in a drive within the industry to develop antibodies with enhanced clinical efficacies and one way to do this is to improve therapeutic antibody glycosylation patterns.

### 2.1 Afucosylated therapeutic antibodies

ADCC is a mechanism by which a number of mAbs mediate their immunotherapeutic action. mAbs that target tumor‐associated antigens and employ ADCC as their sole mechanism of action are still able to yield therapeutic benefit (Hubert et al., 2011). Interestingly, the removal of a core fucose residue from the *N*‐glycan attached to the conserved Asn297 of the Fc region of IgG is known to enhance ADCC activity in vivo by up to 50‐fold (Shields et al., 2002; Shinkawa et al., 2003). This upregulation is a result of the afucosylated IgG having increased affinity for the ADCC‐activating receptor, FcγRIIIa. This discovery stimulated research focused around generating cell lines yielding afucosylated mAbs, as well as investigations into upstream control strategies to direct mAb glycosylation towards particular glycan profiles. As a result, afucosylated mAbs are now licensed as therapeutics in their own right, with examples of such mAbs currently in the market or in clinical trials including obinutuzumab, mogamulizumab, MEDI‐551, DI‐B4, ublituximab, imgatuzumab, and tomuzotuximab (Zahavi et al., 2018).

Rituximab is a CD20‐targeting mAb that was first approved by the FDA for the treatment of relapsed or refractory non‐Hodgkin's lymphoma. It is now a standard treatment for the majority of B‐cell neoplasms either in combination with chemotherapy or as a standalone therapy. Although it has shown good clinical efficacy, evidence that some tumors develop resistance has been reported. In an attempt to mitigate this, obinutuzumab was developed. Among other modifications, obinutuzumab has reduced core fucosylation and therefore increased ADCC activity compared with its fucosylated counterpart rituximab. In the context of chronic lymphocytic leukemia (CLL), obinutuzumab demonstrates strong clinical advantages over rituximab and enhanced progression‐free survival of patients receiving obinutuzumab therapy compared with rituximab (Freeman & Sehn, 2018). Another afucosylated mAb that targets CD20 is ublituximab, which has recently been granted Fast Track Designation by the FDA for the treatment of CLL in combination with umbralisib.

For a number of reasons, efforts towards the production of afucosylated IgG have mainly focused on cell line engineering. First, the application of specific fucosidases for hydrolysis of the core fucose is usually not successful due to steric hindrance. It is also not possible to use size‐, charge‐, or hydrophobicity‐based separation methods due to the fucose having a very low molecular weight and no associated charge. Finally, the use of lectin affinity chromatography (LAC) is not feasible for the isolation of afucosylated mAbs due to specificity issues and potential lectin‐induced toxicity.

### 2.2 Matching glycosylation patterns during mAb biosimilar development

Biosimilars create pricing pressure and competition within the biologics market, which is important for widening patient access to treatments by lowering the cost of healthcare. Over the past few years, there has been considerable development and investment in the biosimilars space as a result of patents expiring for many blockbuster mAbs, such as trastuzumab, rituximab, and adalimumab. Developers of biosimilars benefit from truncated approval procedures that utilize existing knowledge of the reference drug. This means that drug safety and efficacy can be extrapolated, to a certain extent, from the reference drug clinical trial data, which saves a significant amount of time and money.

However, mAbs are large, complex molecules with a variety of factors influencing their primary, tertiary, and quaternary structure. As a result of this complexity, it is difficult to develop cell lines and manufacturing processes such that the required degree of “similarity” to the innovator molecule is achieved. Even small differences in process parameters, media composition and expression systems can result in significant differences in mAb glycosylation between innovator molecules and biosimilars, making it challenging to gain regulatory approval (Duivelshof et al., 2019). As such, biosimilar developers are expected to structurally characterize both the innovator molecule and their proposed biosimilar, including the primary structure, higher or lower molecular weight derivatives of the product, and posttranslational modifications such as glycosylation. In the case of mAbs that elicit effector function for their mechanism of action, varying the *N*‐glycan structure at position Asn297 in the Fc region can confer different ADCC and CDC activities in vivo, and consequently different clinical efficacies. For these reasons, thorough characterization of a mAb's glycosylation profile is essential at all stages of development and during manufacturing. This gives rise to a need for the development of novel, orthogonal analytical techniques for glycoprofile characterization (Duivelshof et al., 2019).

Since the patent for rituximab expired in 2013 in Europe and 2018 in the United States, manufacturers have focused on developing biosimilars (Subramanian et al., 2017). A number of studies have reported the characterization of the glycosylation profiles of rituximab biosimilars and the impact of different glycosylation patterns on the drug's clinical efficacy (Cerutti et al., 2019; D'Atri et al., 2017; Kang et al., 2020; Nupur et al., 2018). In addition to strategies for directing mAb biosimilar glycosylation towards profiles closer to that of the innovator molecule, analytical and structural characterization techniques that can accurately define such profiles are important. In general, liquid chromatographic techniques such as hydrophilic interaction liquid chromatography and ultrahigh‐performance liquid chromatography coupled to mass spectrometry are used to characterize and compare biosimilar glycosylation to the innovator molecule (D'Atri et al., 2017; Kaur et al., 2021).

### 2.3 The impact of the choice of host cell line on glycosylation

The choice of the host cell expression system for human IgG production is mainly restricted by the expression system's ability to produce safe human‐like glycosylation. Most FDA‐ and EMA‐approved biosimilars are mAbs expressed in mammalian systems to avoid generation of glycoepitopes giving rise to allergic reactions or severe immune responses. Besides achieving high yields of authentic and “safe” product with the highest therapeutic activity, other factors such as development and manufacturing costs are considered such as choice of the host cell system, cost and time of purification strategies, and final product yield.

To date, three mammalian cell lines have been widely utilized for glycosylated IgG production: Chinese hamster ovary (CHO), the mouse SP0/2, and the NS0 cell lines, with CHO cells by far the most commonly used for marketed products. Indeed, IgG production in CHO cell lines is well established and the range of glycoproteins produced using this expression system has been recently reviewed (Donini et al., 2021). CHO cells have the potential to present nonhuman terminal Gal‐α1,3‐Gal‐GlcNAc glycoepitopes in the antennary region, which has been associated with severe immune consequences in humans. In 2008, the chimeric mouse–human IgG1 cetuximab caused severe hypersensitivity in 26 of 76 patients receiving cetuximab treatment. The recognition of terminal Gal‐α1,3‐Gal glycoepitopes in the Fab region of the antibody occurred via IgE and induced anaphylaxis. It is assumed that Fab *N*‐glycans are exposed in a preferred steric orientation for IgE binding (Chung et al., 2008).

Sialic acid is primarily connected as Neu5Ac α2,3‐linkages in CHO cell lines. Genomic sequencing of CHO‐K1 cells determined the presence of a ST6Gal sialyltransferase homolog but did not detect any expression of the enzyme (Xu et al., 2011). Human serum IgG *N*‐glycans (Fab‐ and Fc‐region) contain about 10% sialylated structures, the vast majority of which are α2,6‐Neu5Ac‐linkages (Adamczyk et al., 2014). Furthermore, Neu5Gc may be inserted into the antennae of recombinant glycoproteins of CHO cell products, which are considered less desirable, as humans have lost the ability to generate Neu5Gc from Neu5Ac. All humans contain low levels of anti‐Neu5Gc antibodies, up to 0.25% of total serum IgG (Tangvoranuntakul et al., 2003). Therefore, the amount of Neu5Gc glycoepitopes should be kept to a minimum (Ghaderi et al., 2010). Mouse and CHO cell lines exhibit similar issues regarding Gal‐α1,3‐Gal epitopes. Further, NS0, CHO, and Sp0/2 cell lines can generate glycoproteins with Man_5_GlcNAc_2_ high‐mannose type *N*‐glycans. Literature regarding IgG‐Man5 remains controversial. For example, the pharmacokinetic study by Falck et al. (2021) determined pharmacokinetic effects of modified glycoengineered IgG1 derived from CHO cells. To generate large amounts of the desired IgG1 glycoforms, chemo‐enzymatic modification and/or glycosidase treatment were applied. Three engineered IgG1 variants had the following differences in their glycosylation: biantennary agalactosylated complex *N*‐glycan (IgG1‐G0F) as a reference standard, IgG1‐G0F truncated by one *N*‐acetylglucosamine (GlcNAc) (IgG1‐(G0F‐1GlcNAc)), and IgG1‐Man5, and these were injected into individual animals. The clearance rates for IgG1 glycoforms from rat serum were determined to be 1.8‐ to 2.6‐fold quicker for IgG1‐Man5 structures compared with the IgG1‐G0F. IgG1‐(G0F‐1GlcNAc) had a 1.2‐ to 1.4‐fold increase in clearance rate compared with the IgG1‐G0F standard (Falck et al., 2021). Following the previous study, the study by Yu et al. (2012) demonstrated threefold faster clearance rates for IgG1‐Man5 variants compared with complex IgG1‐G0F/G1F structures in mice. The authors also investigated the ability to induce ADCC in vitro using peripheral blood mononuclear cells from healthy blood donors as effector cells in the presence of B cells (WIL2‐S). The EC_50_ value for the induction of ADCC showed a five‐ to sevenfold increase for IgG1‐Man5 compared with the reference IgG‐G0F/G1F mixture. However, IgGs with afucosylated glycoforms were more potent than IgG1‐Man5. Also, the FcgR3a‐binding activity to IgG1‐Man5 had EC_50_ values six‐ to eightfold higher compared with the reference IgG1‐G0F/G1F (Yu et al., 2012). Overall, the literature on pharmacokinetic in vivo studies remains contradictive regarding IgG‐Fc *N*‐glycans role and underlines the field's challenge. Studying clearance rates of IgG‐Fc‐*N*‐glycan variability requires analytical expertise and sensitive techniques to determine glycopeptide sequences and abundancies to exclude Fab glycosylation or other glycovariants. Pharmacokinetic studies are often dependent on multiple factors, such as the study design, manufacturing approach affecting distribution of IgG glycoforms impacted by cell culture feeding conditions, or variation in animal model versus samples derived from human clinical trials. Recent literature highlights that administration parameters influence most animal studies as follows: administration route, the concentration of IgG administered, and collection time points (Chen et al., 2009; Goetze et al., 2011; Yu et al., 2012). However, it is known that oligomannose structures in circulatory systems are cleared by mannose‐binding proteins (C‐type lectins) present on sinusoidal endothelial cells and Kupffer cells with macrophagic activity residing in the liver (Bhandari et al., 2020; Loke et al., 2016). Potential clearance of carbohydrates via mammalian sugar‐binding receptors was reviewed by Taylor and Drickamer (2019).

## 3 UPSTREAM STRATEGIES TO CONTROL GLYCOSYLATION

Attempts to control and direct biologic glycosylation towards certain profiles have primarily focused on cell line engineering and bioprocess control, including process parameter alterations such as pH shifts and the addition of supplements to cell culture media during mAb production.

### 3.1 Cell line engineering strategies

**Figure 4:**
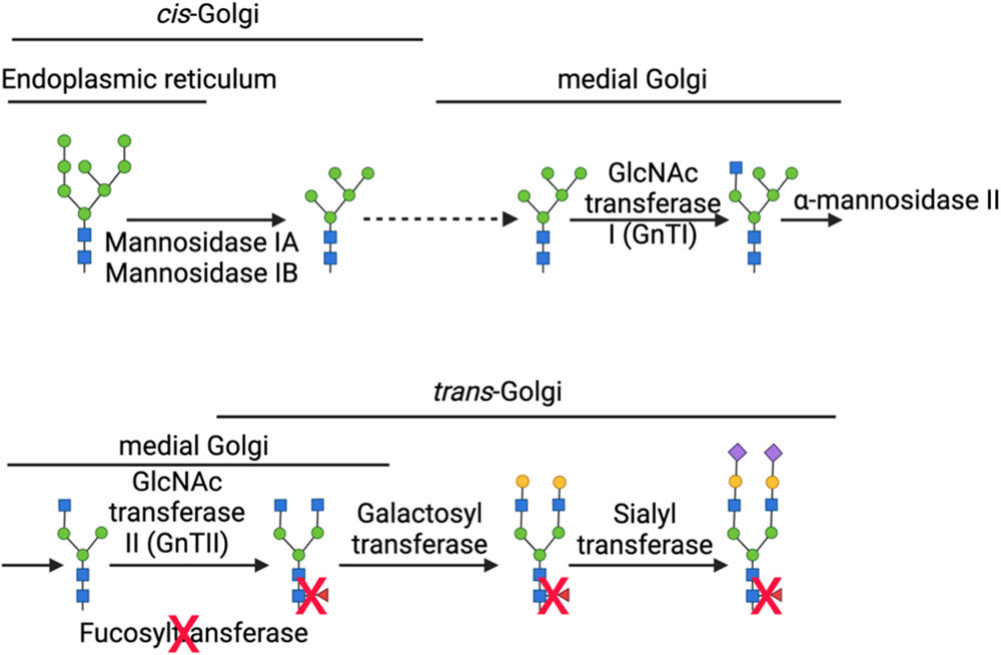
Monoclonal antibody (mAb) glycosylation pathway in Chinese hamster ovary (CHO) cells with the *FUT8* gene knocked out. This engineered pathway yields IgG mAbs with afucosylated glycans attached to Asn297. Created with BioRender.com

Various developments in cell line engineering for mAb manufacturing have aimed to reduce the core fucosylation of the *N*‐glycan at Asn297 in the Fc region of IgG, as this is known to confer the most significant increase in ADCC activity of IgG mAbs compared with galactosylation, mannosylation, sialylation and *N*‐acetyl‐glucosamine levels. The Potelligent® technology was first developed by Kyowa Hakko Kogyo Co. Ltd in 2004 to address this issue. After the discovery was made, which reduced core fucosylation of IgG mAbs results in enhanced ADCC in vivo, the group at Kyowa Hakko Kogyo focused on generating a mammalian cell line that was capable of yielding IgG with reduced core fucosylation to enhance its therapeutic efficacy. The group generated a CHO cell line, which had the gene encoding the fucosyltransferase (ɑ1,6‐FucT) responsible for adding the fucose to the core GlcNAc, *FUT8*, knocked out (see Figure 4). This was achieved by disrupting both *FUT8* alleles using sequential homologous recombination (Shinkawa et al., 2003). These *FUT8* ^−/−^ cells produce IgG, which does not possess core fucose and therefore elicits a greater level of ADCC compared with other fucosylated mAbs. An example of a current therapy manufactured using the Potelligent® CHO *FUT8* ^−/−^ cell line is mogamulizumab, commercially known as Poteligeo®. This mAb was originally approved for the treatment of chemokine C receptor 4 (CCR4)‐expressing T‐cell leukemia–lymphoma and peripheral T‐cell lymphoma (Evans & Syed, 2014). The EMA now list Poteligeo® as a treatment for mycosis fungoides and Sézary syndrome. Another technique that has been used to yield afucosylated IgG mAbs is interference with the synthetic pathway that gives rise to the fucose precursor or substrate for ɑ1,6‐FucT, guanosine diphosphate (GDP)‐fucose. One such approach has been to disrupt the gene that encodes the enzyme GDP‐mannose 4,6‐dehydratase (GMD). GMD is involved in catalyzing the conversion of d‐glucose to GDP‐fucose and is therefore crucial for the activity of ɑ1,6‐FucT in carrying out fucosylation downstream in this pathway. CHO cells deficient for GMD reportedly completely lack GDP‐fucose, and when not provided with sources of l‐fucose in the growth media these cells yield 100% afucosylated IgG (Kanda et al., 2007).

**Figure 5:**
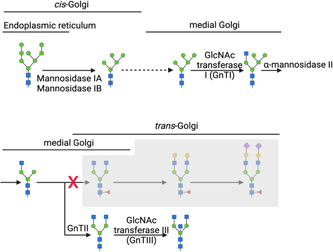
Monoclonal antibody (mAb) glycosylation pathway of Chinese hamster ovary (CHO) cells when diverted towards *N*‐acetylglucosamine (GlcNAc) bisection. The overexpression of β1,4‐*N*‐acetylglucosaminyltransferase III (GnTIII) generates bisected glycans, which cannot be fucosylated due to the steric hindrance created by the GlcNAc residue for c1,6‐FucT. Created with BioRender.com

Another approach to achieving afucosylation is by genetic engineering so that glycans with bisecting GlcNAc are produced. GlcNAc bisection creates steric hindrance for ɑ1,6‐FucT, thereby preventing core fucosylation on resulting glycan structures (see Figure 5). This has been achieved using different approaches, one of which has been to engineer cells to overexpress the enzyme β1,4‐*N*‐acetylglucosaminyltransferase III (GnTIII), which adds a bisecting GlcNAc residue onto the βMan of the *N*‐glycan core structure (Hossler et al., 2009). This technology, commercially known as GlycoMAb®, was developed by Glycart (Evans & Syed, 2014). GlycoMAb® technology has since been optimized so that the localization motif of the Golgi ɑ‐mannosidase II is incorporated into the expressed GnTIII. This results in the GnTIII enzyme outcompeting ɑ1,6‐FucT more effectively (Ferrara et al., 2006), possibly due to the fact that the addition of the localization motif means that the GnTIII is more likely to act earlier in the glycosylation pathway when core fucosylation has not yet occurred, therefore preventing fucosylation more successfully (see Figure 3). This particular platform is used to produce obinutuzumab (Yu et al., 2017).

Alternatively, reduction in the level of core fucosylation can be achieved by engineering CHO cells to heterologously express the enzyme GDP‐6‐deoxy‐d‐lyxo‐4‐hexulose reductase (RMD). As described above, this mechanism also affects the GDP‐fucose synthesis pathway, but this time indirectly. RMD is responsible for the production of GDP‐*D*‐rhamnose, which negatively inhibits the GMD enzyme. By inhibiting GMD, the production of GDP‐fucose is downregulated and the pool of substrate for the αɑ1,6‐FucT enzyme is depleted. This approach has shown remarkable success, yielding 98% afucosylated IgG (von Horsten et al., 2010). The platform was developed by ProBioGen and is commercially known as GlymaxX® technology, which was licensed to Bayer in 2019. Bayer stated that they would “leverage the technology to further increase the potency of an undisclosed antibody candidate for oncological indications” (BioSpace, 2019).

The humanization of the yeast *N*‐glycosylation pathway was a significant breakthrough in cell line engineering. A review of early efforts in this space is provided by Wildt & Gerngross (2005), which covers the first attempts to engineer *Saccharomyces cerevisiae* in the 1990s and some of the studies carried out in the early 2000s. Choi et. al (2003) reported engineering of the *Pichia pastoris* glycosylation pathway so that it could perform some early human *N*‐glycan processing steps. First, the gene encoding α1,6‐mannosyltransferase, the initiating enzyme in the *P. pastoris* glycosylation pathway, was deleted. Several combinatorial genetic libraries were then constructed using fungal type II membrane proteins and the catalytic domains of enzymes from mammals, insects, amphibians, worms, and fungi with the aim of generating αɑ1,2‐mannosidase and human β1,2‐*N*‐acetylglucosaminyltransferase I (Choi et al., 2003; see Figures 2, 3, and 4). Further investigation has revealed more enzymes within the yeast glycosylation pathway that must be targeted and deleted to reduce hypermannosylation, as well as how best to localize active forms of enzymes downstream in this pathway required for converting the Man_8_GlcNAc_2_ to Man_5_GlcNAc_2_, then to GlcNAcMan_5_GlcNAc_2_ and so on (see Figures 3 and 4). A review of these efforts is provided by Laukens et al. (2015). Another recent study used CRISPR/Cas9 to humanize the yeast *Kluyveromyces marxianus* (Lee et al., 2020). The success of these efforts suggest that some IgG biotherapeutics could eventually be produced using yeast expression systems.

The degree and type of sialylation of mAbs can also be altered by engineering the host expression system. In one study in CHO cells, the enzyme that attaches sialic acid in an ɑ2,6 linkage was overexpressed to increase the amount of αɑ2,6 sialylation and CRISPR/Cas9 was used to disrupt the genes encoding the enzymes responsible for ɑ2,3 sialylation, thus reducing levels of αɑ2,3 sialylation. This is favorable, as ɑ2,6 sialylation in the Fc region of IgG has been reported to increase ADCC activity compared with ɑ2,3 sialylation (Chung et al., 2017).

Despite the success of cell line engineering strategies for altering the glycosylation of IgGs, development has been arduous and expensive. There are often significant regulatory hurdles that must be overcome when gaining approval for the use of a new cell line for biologics manufacturing, such as demonstrating the required product quality. Combining this with the fact that not all genetic engineering strategies have yielded overwhelming levels of success, research efforts have also focused on fermentation process parameter alterations, media supplementation and enzymatic glycoengineering.

### 3.2 Upstream processing conditions

Manipulating fermentation process conditions is arguably a cheaper and possibly less time‐consuming approach to controlling mAb glycosylation than cell line engineering and can be applied during a bioprocess to tune glycan patterns. A number of groups have experimented with pH changes in particular; low pH has commonly been found to enhance galactosylation and sialylation, but concomitantly decreases specific productivity (Aghamohseni et al., 2014). Galactosylation is generally associated with an increase in CDC activity, whereas sialylation is known to result in increased serum half‐life and ADCC (St. Amand et al., 2014). In a study investigating the effect of temperature shifts on glycosylation macroheterogeneity, a reduction in culture temperature from 37°C to 33°C increased glycosylation site occupancy of recombinant human tissue plasminogen activator by up to 4% and an increase in culture pH resulted in a decrease in site occupancy (Gawlitzek et al., 2009). Temperature reductions in fermentation have had differing effects on glycan structures, depending on the system that is being experimented with; in some cases, more processed glycan structures are generated and in other cases less so (Villiger et al., 2016).

### 3.3 Media supplementation

Media supplementation approaches have shown greater utility to yield different glycoforms. This approach can be useful for directing glycosylation towards certain profiles, for example, increasing sialylation through the addition of *N*‐acetyl‐mannosamine (ManNAc) (Yin et al., 2017) or manganese, galactose, and uridine (Villacrés et al., 2021) to the culture medium. As with cell line engineering, many media supplementation approaches have focused on reducing IgG Fc core fucosylation due to this having the most significant impact on ADCC activity out of all known oligosaccharide variations. A common addition to cell culture media when attempting to yield afucosylated IgG is kifunensine, an inhibitor of ɑ1,2‐mannosidase I. This enzyme is responsible for trimming the *N*‐glycan to a form that can be subsequently fucosylated. Other additions to cell culture media which have been investigated for preventing fucosylation are 2‐deoxy‐2‐fluorofucose and 5‐alkynylfucose, which are both inhibitors of the fucosyltransferase αɑ1,6‐FucT. Although addition of these inhibitors to cell culture media have been shown to reduce IgG Fc core fucosylation, results from one study showed that there was significant incorporation of these analogs into the resulting IgG glycans and 2‐deoxy‐d‐fluorofucose in particular is known to be toxic. The group stated they had not investigated the effect that the incorporation of these analogs into mAb glycans had on potency and safety (Zimmermann et al., 2019).

Other media supplementation approaches have been studied with the aim of targeting biologic glycosylation more generally, including degrees of mannosylation, galactosylation GlcNAcylation, and sialylation. The addition of MnCl_2_ can direct glycosylation towards more highly mannosylated and galactosylated structures, which is likely to enhance mAb ADCC activity. MnCl_2_, uridine, and galactose supplementation has been shown to increase galactosylation levels, as can the addition of glutamate (but not glutamine) to cell cultures (St. Amand et al., 2014). The effect of differing levels of ammonia has been investigated by several groups, with the consensus being that in the case of mAbs, higher ammonia concentrations reduce glycosylation overall. However, many reports are contradictory and depend on other factors such as the cell line, glucose, lactate, and amino acid concentrations, dissolved oxygen and shear rate (Aghamohseni et al., 2014). This is yet another reflection of how sensitive glycosylation pathways are to process factors and the difficulty in its control.

Due to its impact on ADCC activity, the levels of mannosylation of IgG biosimilars must be monitored carefully. High mannose structures (Man5 and higher) have been reported to increase as a result of the addition of alternative sugars and amino sugars to the culture medium when producing a mAb using a CHO cell line, although upon scale‐up this supplementation caused a secondary interaction with osmolality. Mannose and glucosamine in combination with either fructose, galactosamine, or both fructose and galactosamine resulted in increased high‐mannose levels of the biosimilar compared with the innovator molecule in the region of 140%–160%, which correlated with an increased ADCC activity (>100%) compared with the innovator molecule (Rameez et al., 2021). As well as increasing ADCC activity, high mannose structures in the Fc region of IgG are also commonly associated with a faster serum clearance rate compared with other glycosylation structures in this region (Goetze et al., 2011; Yu et al., 2012), making mannosylation an important process to control during antibody production. Ornithine, spermine, and copper in fermentation media have been found to correlate with levels of high mannose, which is postulated to be via the polyamine pathway and cellular redox (Kang et al., 2015). Kang et al. (2015) also noted that conditions of low osmolality resulted in decreased levels of high mannose, which had been found in previous studies (Pacis et al., 2011). Conversely to the effect of increasing manganese concentration, which results in reduced mannosylation, depriving the culture of copper has been found to reduce levels of high mannose glycans. When high mannose levels are high, ornithine accumulates in the culture medium. Supplementation of the media with ornithine also results in increased levels of high mannose and reduction of spermine in the media results in reduced high mannose levels. Spermine is an inhibitor of the enzyme that converts ornithine into putrescine in the polyamine pathway; by lowering the amount of spermine, ornithine accumulates and, therefore, the levels of high mannose increase (Kang et al., 2015). These results suggest a link between the polyamine pathway and glycosylation, although this has not been studied extensively.

A design of experiments approach was used by Loebrich et al. (2019), to evaluate the effect of commonly used media additives to control glycosylation of an IgG1 produced in a CHO‐K1 cell line. They found that 12.5 mM glucosamine decreased galactosylation levels, 5.0 mM uridine increased galactosylation, and 1.0 mM copper decreased levels of Man5 glycan structures. The degrees to which these supplements achieved these effects varied, with some having concomitant effects on other glycan structures; 1.0 mM copper, for example, also increased levels of G0, G0F, and G1F (glycans with no galactosylation, no galactosylation and one fucose, and one galactose and one fucose, respectively). The effect of other supplements, such as glycerol, cytidine, and ManNAc, was also investigated, the latter of which at lower concentrations increased levels of G0F specifically but did not affect the levels of other glycan structures (Loebrich et al., 2019).

## 4 DOWNSTREAM STRATEGIES TO CONTROL GLYCOSYLATION

A complementary or alternative approach to glycan tuning is to develop and utilize downstream processing techniques that can be used in conjunction with upstream processing technologies or as standalone techniques in the manufacturing of biologics with specific glycosylation patterns, such as afucosylated mAbs.

### 4.1 Enzymatic remodeling

A number of studies have investigated the use of endoglycosidases for antibody glycan remodeling. EndoS is an endoglycosidase that cleaves between the two GlcNAc residues of the *N*‐glycan core. It is specific for IgG and has been used for the remodeling of IgG Fc *N*‐glycans in a number of studies (Kurogochi et al., 2015; Li et al., 2018; Sjögren et al., 2020). The discovery of this enzyme sparked interest in this area, including the immobilization of WT EndoS and its glycosynthase mutant D184M with gained transglycosylation activity for the enzymatic glycoengineering of IgG (Li et al., 2018). Other endoglycosidases are specific for different oligosaccharide structures. EndoH, for instance, cleaves high‐mannose and hybrid *N*‐glycans, and has been used for glycoprotein remodeling, whereas EndoD cleaves the GlcNAc‐β1‐4‐GlcNAc linkage in the chitobiose core region of *N*‐glycans, resulting in a truncated β‐GlcNAc‐α1,6‐Fuc on a glycoprotein (Sjögren et al., 2020). Enzymatic remodeling techniques have shown significant potential in vivo for generating IgG with more homogenous glycoprofiles. However, when it comes to yielding afucosylated IgG, enzymatic techniques have their limitations. As mentioned in Section 2.1, fucosidases are not efficient in removing fucosylated structures from IgG due to steric hindrance.

During glycan remodeling reactions, many endoglycosidases utilize sugar oxazoline transition state analogs, which act as the glycan donor substrates for transglycosidases (Yang & Wang, 2017). However, oxazoline compounds can undergo side reactions if conditions are not controlled within strict limits; the carbon between the nitrogen and oxygen of oxazoline can undergo nucleophilic attack from a reactive amino group, such as a lysine side chain. These side reactions are undesirable, particularly when manufacturing clinical‐grade material, as they can make downstream operations more complex and alter product function (Manabe et al., 2018).

For these reasons, developments have been made to negate the need for the sugar oxazoline donor substrate for glycan remodeling. Many of these efforts have focused on engineering endoglycosidases and using alternative glycan donors (Boune et al., 2020). The glycosylation state of trastuzumab has been remodeled using mutant forms of EndoS, EndoM, and endo‐CC. In one study, an EndoM mutant was used to remove a fully sialylated glycan from sialylglycopeptide (SGP), and an EndoS mutant with transglycosylation activity then catalyzed the addition of this glycan to the Fc region of deglycosylated trastuzumab (Iwamoto et al., 2018). In another study, the use of an oxazoline donor substrate was successfully negated by using SGP as the donor and EndoS to deglycosylate an anti‐CCR4 antibody followed by a mutant endoglycosidase, endo‐CC N180H, to transfer the glycan from SGP to the antibody (Manabe et al., 2018). Although particular mutants of these endoglycosidases achieved high efficiency in these studies, and this method would likely increase the glycosylation heterogeneity of other IgG mAbs, the addition of further steps during larger‐scale mAb processing is likely to be unattractive to manufacturers due to the inevitable reduction in yield that these extra processing steps would cause. In addition, this method requires the presence of a donor glycoprotein; in the case of the work carried out by Manabe et al. (2018), it was noted that a particularly large amount of SGP donor substrate was required for the successful transglycosylation reaction. This adds cost to a process and can complicate downstream processing.

### 4.2 Chromatographic separations

Although enzymatic remodeling efforts have yielded success, their use is rarely reported at larger scales. Developments have been made in the area of chromatography that focus on glycoform separations, such as the use of anion or cation exchange chromatography to separated out sialylated species. This is possible due to the negative charge on terminal sialic acid residues and is a technique that has primarily been used at an analytical scale (Hurum & Rohrer, 2011; Rohrer, 2000). This section of the review focuses on affinity ligand development that has been made towards separating differentially glycosylated recombinant proteins.

#### 4.2.1 Lectins

One chromatographic technique that has gained interest over the past decade has been LAC. Lectins are proteins or glycoproteins often derived from plants which have the ability to bind specific carbohydrate moieties, making them useful ligands for separating different glycoforms of glycopeptides, glycoproteins, or glycolipids (Hage et al., 2012). Although monomeric binding affinities of lectins can be low, multivalent interactions can increase binding affinities into the nanomolar range and the binding is reversible, which are characteristics consistent with properties that make for suitable affinity ligands. Lectins do not react with or modify their targets and generally remain stable at conditions of variable pH and ionic strength, meaning that their three‐dimensional structure and binding affinity for their target is unlikely to alter after elution or after several chromatographic cycles. Lectins have been successfully immobilized onto Sepharose, silica beads, and some monolithic supports, with no significant perturbations in binding affinity reported.

Despite the suitability of lectins for use as affinity ligands and their ability to separate different product glycoforms, they are not widely used, particularly in industrial‐scale processing. This is probably because of difficulties in consistent manufacturing, as lectins are often multimeric with many glycosylation sites themselves, making them difficult to produce recombinantly. This results in batch‐to‐batch variation, which compromises their effectiveness as affinity ligands (O'Connor et al., 2017). There is also the potential for these resins to leach toxic lectins (Bolton et al., 2013). In addition, eukaryotic lectins often require further posttranslational modification, which not only increases their structural complexity but also renders them unsuitable for recombinant production in a bacterial host. Attempts have been made to synthesize plant lectins recombinantly, but these efforts have normally resulted in very low yields of insoluble protein. These issues have made lectin production almost impossible to scale up and has constrained their use to analytical‐scale applications (Keogh et al., 2014). In addition, in the context of enriching for afucosylated IgG, the use of lectins is not suitable. Although one study did identify the ability of Aleuria Aurantia Lectin to specifically bind fucose moieties on IgG, this lectin is only capable of doing so if the antibody is denatured or treated with a glycosidase (Chen et al., 2012).

#### 4.2.2 Fc γ‐ receptors

**Figure 6:**
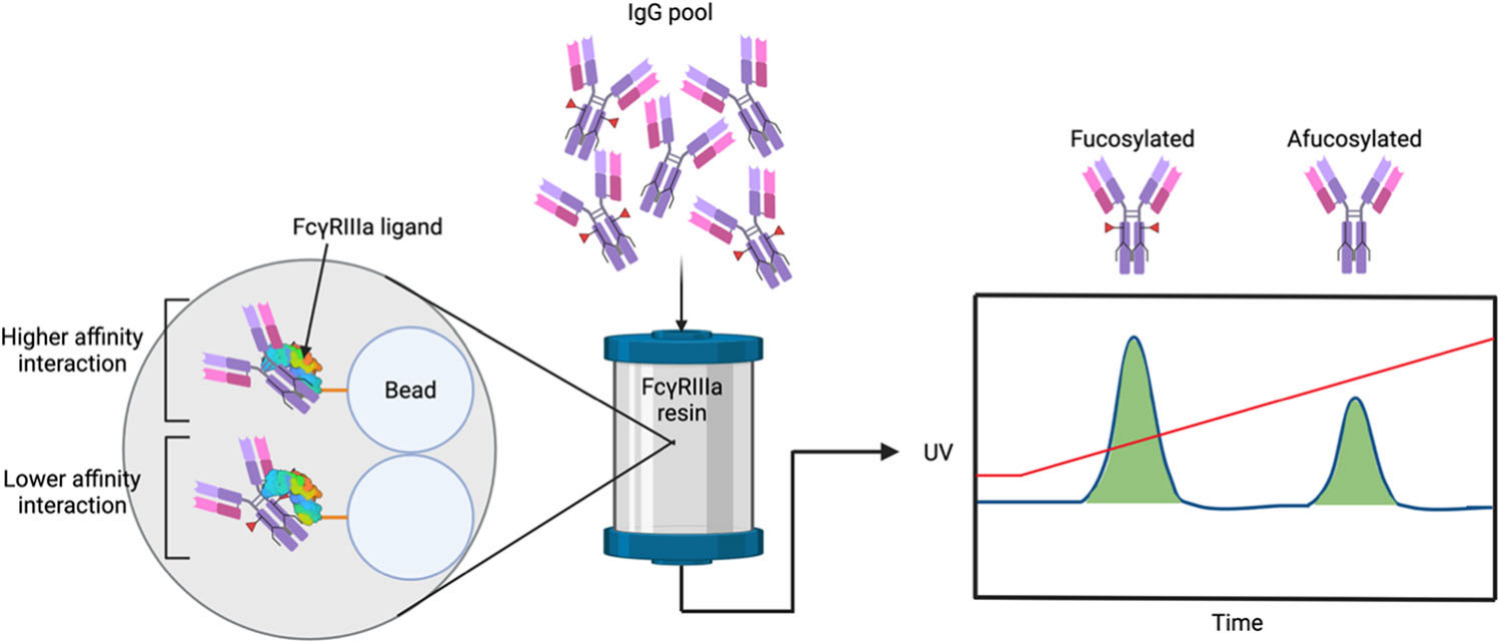
Schematic of the fucosylated and afucosylated IgG glycoform separation afforded by glycosylated FcγRIIIa‐based ligands. The pool of IgG loaded onto the column is heterogeneously glycosylated and a gradient elution (red line) is used. Fucosylated IgG binds the ligands with lower affinity and therefore elutes off the column first. As the elution gradient increases, the more tightly bound afucosylated IgG eventually also elutes. Created with BioRender.com

Affinity ligands based on the FcγRIIIa have been developed and patented in various economic areas for the separation of fucosylated and afucosylated IgG (EP 2 768 845 B1, US 10221210 B2, US 2016/0222081 A1; see Figure 6). The first company to do this was Zepteon, who obtained their patent covering the United States in 2019. This group transiently expressed WT FcγRIIIa in HEK293 cells and immobilized it to an activated N‐hydroxysuccinimide (NHS) Sepharose resin using amine chemistry. These ligands have been successfully used in different settings to enrich for afucosylated IgG (Boesch et al., 2018; Freimoser–Grundschober et al., 2020; Lippold et al., 2019). In particular, they are used during IgG‐type mAb biosimilar development to match fucosylation levels of the biosimilar with the innovator molecule, which is key to obtaining regulatory approval, and during lot‐to‐lot comparisons.

Despite the proven capability of these ligands for enriching afucosylated IgG, the yield obtained has been low and would not be viable in industrial‐scale processing (Bolton et al., 2013). Reasons for this are likely to be due to WT FcγRIIIa possessing up to five glycosylation sites, giving the ligands a large hydrodynamic radius; a variety of highly complex glycans at these sites could affect the overall shape of the ligands and, consequently, their hydrodynamic properties that may influence their separation capabilities. It could also be related to the immobilization chemistry used, as there are a number of lysine residues in the binding site of WT FcγRIIIa. The biocompatible 1‐ethyl‐3‐(3‐dimethylaminopropyl)carbodiimide (EDC)/NHS 1‐ethyl‐3‐(3‐dimethylaminopropyl)carbodiimide/method is neither an orthogonal nor a specifically targeted labeling strategy, so multiple surface‐exposed lysine residues of the WT FcγRIIIa are conjugated to for immobilization. The IgG binding of the WT FcγRIIIa may therefore be compromised, resulting in a lower separation efficacy. The Japanese company Tosoh have developed aglycosylated FcγRIIIa‐based affinity ligands with enhanced pH and/or temperature stability compared with WT FcγRIIIa. Although these ligands are not capable of discriminating between fucosylated and afucosylated IgG due to their lack of glycosylation themselves, they do seem to be able to separate IgG based on galactosylation levels (Kiyoshi et al., 2018).

## 5 FUTURE PERSPECTIVES

As our understanding of glycosylation pathways and the effects of glycosylation on biologic immunogenicity and pharmacokinetics deepens, and indeed as time goes on, the stringency of regulations surrounding mAb glycosylation and the importance of controlling and monitoring it is likely to increase. The upstream approaches to controlling mAb glycosylation have not proved infallible and it is therefore necessary for downstream approaches to be employed in conjunction with upstream technologies to yield products within a desired glycosylation specification. Advancements have been made in the field of chromatographic affinity ligand development, primarily for the separation of fucosylated and afucosylated IgG, but there is ample opportunity for further research in this area to enhance our ability to produce IgGs with much more defined glycosylation profiles. Improvements to online monitoring technologies for product glycosylation status would also be beneficial when seeking regulatory approval for new mAbs, mAb derivatives or biosimilars and attempting to demonstrate lot‐to‐lot consistency.
